# Application of HPTLC Multiwavelength Imaging and Color Scale Fingerprinting Approach Combined with Multivariate Chemometric Methods for Medicinal Plant Clustering According to Their Species

**DOI:** 10.3390/molecules26237225

**Published:** 2021-11-29

**Authors:** Simona Codruța Aurora Cobzac, Neli Kinga Olah, Dorina Casoni

**Affiliations:** 1 Department of Chemistry, Faculty of Chemistry and Chemical Engineering, Babeş-Bolyai University, Arany János, No. 11, RO-400028 Cluj-Napoca, Romania; simona.cobzac@ubbcluj.ro; 2 Research Center for Advanced Chemical Analysis, Instrumentation and Chemometrics–ANALYTICA, Babeş-Bolyai University, 11 Arany Janos Str., RO-400028 Cluj-Napoca, Romania; 3 Faculty of Pharmacy, “Vasile Goldis” Western University of Arad, 86 L. Rebreanu Str., RO-310045 Arad, Romania; neli.olah@plantextrakt.ro; 4 SC PlantExtrakt SRL, RO-407059 Rădaia, Cluj, Romania

**Keywords:** HPTLC, image analysis, color scale fingerprinting, chemometric approaches, medicinal plants

## Abstract

In the current study, multiwavelength detection combined with color scales HPTLC fingerprinting procedure and chemometric approach were applied for direct clustering of a set of medicinal plants with different geographical growing areas. The fingerprints profiles of the hydroalcoholic extracts obtained after single and double development and detection under 254 nm and 365 nm, before and after selective spraying with specific derivatization reagents were evaluated by chemometric approaches. Principal component analysis (PCA) with factor analysis (FA) methods were used to reveal the contribution of red (R), green (G), blue (B) and, respectively, gray (K) color scale fingerprints to HPTLC classification of the analyzed samples. Hierarchical cluster analysis (HCA) was used to classify the medicinal plants based on measure of similarity of color scale fingerprint patterns. The 1-Pearson distance measurement with Ward’s amalgamation procedure proved to be the most convenient approach for the correct clustering of samples. Data from color scale fingerprints obtained for double development procedure and multiple visualization modes combined with appropriate chemometric methods proved to detect the similar medicinal plant extracts even though they are from different geographical regions, have different storage conditions and no specific markers are individually extracted. This approach could be proposed as a promising tool for authentication and identification studies of plant materials based on HPTLC fingerprinting analysis.

## 1. Introduction

The medicinal plants consumed preferably as teas or tinctures have a long and rich history regarding their usage as therapeutics. According to the World Health Organization (WHO) report, in the last decades the interest of population for herbs as a replacement for new innovative drugs has growth considerably due to their lack of side effects and toxicity. According to the British National Formulary, nowadays, 56% of the new therapeutics are started from natural products [1,2,3] and many herbs are also considered a source of natural ingredients used to enhance the aroma, flavor or color of the food, and more importantly, to prevent lipid oxidation and contribute to food preservation [1,4].

In order to use a plant as a herbal medicinal product it is important to ensure its correct identification. The identification is performed observing and comparing the macroscopic properties, meaning the aspect, color, smell of vegetal raw materials respectively the morpho-anatomical properties with the description from the compendial monographs. For therapeutic purposes, the plants can be used entirely or just some vegetative parts. Even though the evaluation of the entire plant could offer more information for identification, this cannot be performed just in exceptional cases because the different parts of plants are collected in a certain period of plants’ development. For better identification, the microscopic analyses of some specific organs and cell constituents could be very helpful and usually are completed by comprehensive fingerprinting approaches.

The hydro-alcoholic extracts from medicinal plants are mixtures of many phytoconstituents that may act individually, additively, or in synergy to improve health and give specific therapeutic effect [5,6]. It is practically impossible within quality control to take account of all constituents, apart from the fact that some of them cannot be analytically detected and hence the complete chemical profile is far from being known. The chemical composition of an extract, reflected by its fingerprint, is specific to a plant from a certain species. Different profiles are obtained for extracts of different parts of the same plant. The geographical growing area (pedoclimatic conditions) and development stage also have a large impact on their chemical composition [7].

Numerous analytical techniques have been developed for medicinal plant identification and quality control analysis [8,9,10,11,12]. To comply with the current regulatory requirements for quality control and labeling, the analysis is based on marker approach, the multicomponent-based approach, and on pattern recognition or fingerprinting approach [13,14,15]. The use of a fingerprint profile is becoming widely accepted as an effective way to describe the complexity of components present in medicinal plant extracts. The fingerprinting concept is ideal for evaluation of identity, authenticity and consistency of herbal products because the fingerprint analysis is able to demonstrate both similarity/uniformity and differences between samples. The World Health Organization (WHO) [16], the American Food and Drug Administration (FDA) [17] and the European Medicines Agency (EMA) [18] have accepted the fingerprint analysis for identification and characterization of herbal drugs and as a strategy for assessing consistency between batches of plant-based medicines. Chromatographic methods, such as high-performance liquid chromatography (HPLC), high-performance thin layer chromatography (HPTLC) and gas chromatography (GC), are commonly used to develop characteristic fingerprint profiles [15,19]. Among the chromatographic methods, HPTLC has been considerably improved in recent years being appreciated in food, biological and pharmaceutical areas [20,21,22,23,24,25]. By the application of powerful scanning instruments, advanced image capturing devices, advanced image processing algorithms and image analysis (IA) protocols, development of novel stationary phases, as well as various separation procedures, HPTLC fingerprinting approaches becoming attractive and fruitful for screening of the complex natural samples [24,26,27,28,29,30,31,32]. In addition to the advantage of multiple parallel sample analysis on the same chromatographic plate and possibility of development in more than one steps using different chromatographic conditions and/or in two directions, HPTLC is the only chromatographic method that offers the possibility to present results as different colorful images by multiple levels of visualization under visible and UV light conditions under 366 nm and/or 254 nm. The use of HPTLC with various detectors is considered a powerful analytical tool especially for the phytochemical applications, such as herbal material analysis, herbal drugs quantification and fingerprint analysis of plants [33]. Combined with image analysis and different chemometric approaches, the HPTLC method was successfully used for quality control and characterization of herbal products and similarity assessment of medicinal plants [34,35,36,37]. In a recent study, the efficacy associated to the information provided by different color scale fingerprints in TLC analysis of complex samples was revealed for correct grouping/classification of a set of medicinal plant extracts [38]. The principal component analysis (PCA) and fuzzy principal component analysis (FPCA) pattern recognition methods were applied for evaluation of data in the chromatographic fingerprinting assays [12,35]. The PCA allows easy visualization of all information contained in a data set by transforming multidimensional data into 2D or 3D coordinates and grouping samples according to similarity and defining variables responsible for certain classification [39]. Hierarchical clustering analysis (HCA) was extensively used to group samples into clusters, based on fingerprint similarity within a class and dissimilarity between different classes, according to a predefined criterion [40]. In the present study, the efficiency of the single/double development combined with multiple visualization modes and color scale fingerprint patterns analysis have been evaluated for the first time for direct clustering of medicinal plants with different geographical growing areas. Using the principal component analysis (PCA) and factor analysis (FA) methods a chemometric evaluation of the individual contribution of information provided by red (R), green (G), blue (B) and, respectively, grey (K) color scale fingerprints have been made. Hierarchical cluster analysis (HCA) has been used to clasify the medicinal plants samples based on measure of similarity of color scale fingerprint patterns. The chemometric analysis of data from all color scale fingerprints using double development procedure combined with multiple visualization modes proved to detect similar medicinal plant extracts even though they are from different geographical regions, have different storage conditions and no specific markers are individually extracted.

## 2. Materials and Methods

### 2.1. Chemicals and Materials

Ethanol, ethyl acetate, toluene and formic acid were purchased from Merck (KGaA, Darmstadt, Germany). Polyethylene glycol (PEG) and anisaldehyde were from Sigma-Aldrich (Steinheim am Albuch, Baden-Württemberg, Germany). Additionally, 2-aminoethyl diphenylborinate (NTS) was purchased from Fluka (Steinheim am Albuch, Baden-Württemberg, Germany). All solvents used for extraction, for mobile phase preparation and plate derivatization were of analytical purity grade.

### 2.2. Plant Material and Extraction Procedure

The study was carried out on thirty-nine medicinal plants belonging to twelve different families and with different European provenience areas (Romania, Macedonia and Hungary) (Table 1).

The plants material originated from North Macedonia was collected from three localities in the Osogovo mountains basin situated in the south-eastern part of the country. They were identified by determination key using the data from Matevski [41] and a specimen is kept in the herbarium at the Department of Plant Production, Faculty of Agriculture, Goce Delchev University in Shtip, Republic of North Macedonia. Samples of Hungarian and Romanian provenience (teas for infusion) were purchased from a specialized store as certified materials assumed in concordance with regulations of Romanian and Hungarian Pharmacopoeias by the producers (Dacia Plant, Fares and Plafar National Company) with a long-standing tradition and positive trend in terms of preparing natural products as well as soils which facilitate green cultures of medicinal and aromatic herbs [42]. A sample of every plant material used in this study is kept at Chemistry Department of Faculty of Chemistry and Chemical Engineering, Babes-Bolyai University, Cluj-Napoca, Romania.

In order to perform the experiment, the vegetal material (10 g) was crushed to powder using a Retsch MM400 ball mill (Retsch, Haan, Germany). Accurately weighted 2 g of each sample were subjected to the maceration process with 20 mL of extraction mixture consisting of ethanol–water in a ratio of 70:30 (*v/v*) for 10 days at room temperature. The resulting extracts were separated by decantation and the remaining residue was washed two times with 2 mL of extraction mixture and centrifuged. In each case the combined extracts were brought to a final volume of 25 mL with the solvent previously used for extraction. The extraction procedure was carried out on two parallel samples in each case.

### 2.3. HPTLC Procedure

Volumes of 30 µL of the hydroalcoholic extracts were applied as 10 mm bands at 10 mm from the bottom edge of the plates, on HPTLC Silica gel 60 F_254_ plates 20 cm × 10 cm (Merck, Darmstadt, Germany) by means of a Linomat V TLC auto-sampler (CAMAG, Muttenz, Switzerland) using a Hamilton syringe and a delivery speed of 80 nL/s.

For a good separation of the extracted phytocompounds, a one-dimensional with double development procedure was applied using a normal chromatographic chamber (CAMAG, Muttenz, Switzerland) which was previously saturated with mobile phase for 30 min at room temperature (≈20°) in each step. In the first development (D1) was performed over a migration distance of 9 cm using the ternary system consisting of ethyl acetate–formic acid–water (80:10:10 *v/v/v*) as mobile phase. The second development (D2) selected according to literature publications [43] was carried out in the same direction after plate drying, using the binary system toluene–ethyl acetate (95:5, *v/v*) as mobile phase and a developing distance of 14 cm. The HPTLC procedure was applied for duplicate chromatographic plates.

Multiwavelength detection at 254 nm and 365 nm was carried out after first and second development. Additionally, after the second development, the plates were selectively sprayed with specific derivatization reagents. Thus, the compounds separated in the first part of the plate (10 cm from the bottom of the plate) were visualized by using 2-aminoethyldiphenylborate solution (NTS—0.2% in ethanol) and PEG while the compounds separated after the second development (the portion of the plate between 10 and 15 cm) were visualized by spraying with anisaldehyde solution. Plate evaluation was performed under 365 nm excitation wavelengths in the fluorescent emission mode. Image of the chromatographic plate was acquired using a Reprostar 3 (CAMAG, Muttenz, Switzerland) system. The HPTLC analysis was carried out using two parallel extracts for each sample and duplicate chromatographic plates. 

### 2.4. Image Processing

TLC Analyser digital scanning software (Version 1.1) was used for image processing and digitised chromatogram data acquisition (http://www.sciencebuddies.org/science-research-papers/tlc_analyzer.shtml, accessed on 10 July 2021). This program virtually plans across the obtained JPG image acting as a simulated TLC scanner that assumes scanning the surface of the chromatographic plate along with the developed track. The following parameters were selected for scanning process: eye dropper size 5 × 5; left margin 45; right margin 720 for the first development and 300 for the second development and scan row from 85 to 585 with a rising increment of 100 (these values correspond to the middle of the application bands). At each measurement point, the intensity of the reflected light is recorded and finally all of the measurements form the densitograms describing changes in the optical density and intensity of the signal along each line. Image density values for pure RGB color (red, green and blue channel) and, respectively, for black and white image (K, grey channel) were plotted by multispectral scan in order to provide corresponding color scale fingerprints. The numerical values obtained by digitization of the individual RGB and K spectral scans were further used as initial variables in the chemometric analyses. 

### 2.5. Chemometric Analysis of Data from Color Scale Fingerprints

Principal component analysis (PCA) combined with Factor Analysis (FA) method and Hierarchical cluster analysis (HCA) were used as multivariate exploratory techniques of data provided by digitization of individual color scales fingerprints. FA with varimax rotation algorithm was used to extract the most relevant information from each color scale fingerprint. The first principal components (PCs) obtained by PCA analysis of data from individual color scale fingerprints (red, green, blue and gray scales) were used as initial variables in FA analysis. Factor loadings values were used to reveal the individual contribution of each color scale fingerprint to the chromatographic profile characterization of the medicinal plants extracts.

Hierarchical cluster analysis (HCA) with joining tree clustering algorithm was used to clasify the selected extracts by join toghether samples into succesively larger clusters based on some measure of similarity. Different clustering distance measurements (Euclidean, Squared Euclidean, City-block (Manhattan), Chebychev, Power, Percent disagreement and 1-Pearson r) and different linkage or amalgamation rule (including Single linkage (nearest neighbor), Complete linkage (furthest neighbor), Unweighted pair-group average (UWPGA), Weighted pair-group average (WPGA), Unweighted pair-group centroid (UWPGC), Weighted pair-group centroid (WPGC) and Ward’s method) were applied in order to evaluate their efficiency in correct classification of the samples. The initial data matrix in HCA analysis (39 cases (samples) and x 17941 variables (from both 254 nm and 356 nm detection and all color scale fingerprints)) was composed of numerical values (as independent variables) related to intensities of separated chromatographic bands corresponding to well-defined R_F_ values and calculated according to the total units considered from the start to front of the plate.

For chemometric analysis of results, Statistica 8.0 (StatSoft, Inc. 1984–2007, Tulsa, OK, USA) software package was used.

## 3. Results and Discussion

### 3.1. Analysis of the HPTLC Chromatograms

Medicinal plant extracts are complex mixtures of different types of phytocompounds such as polyphenols, terpenes, carotenoids, steroids, and others. The chemical composition of the extracts depends on the plant species (interspecies variability) and extraction system. Different parts of the same plant (root, fruits, flowers, stems or leaves) have different and/or large variability of chemical composition and the analysis methods or marker-based identification have become very complex especially due to the presence of other many variables, including location (intra-species variability) and time of collection and harvesting. In these cases, the identification procedure based on the potential physical, chemical and biochemical similarities/differences between samples is difficult to perform [7]. The use of the chromatographic fingerprint profile is accepted as an effective way to describe the complexity of components present in medicinal plant extracts. In the simplest way, identification of plant material is performed by comparing the chromatographic profile with that of reference samples (certified botanical sample). Therefore, according to the aim of the present study, the extraction system was selected to extract as many as possible classes of compounds with a wide polarity range and provide a complex chromatographic profile. As recommended by the European Medicines Agency [44], ethanol–water mixture (70% ethanol) is widely used to obtain tinctures from medicinal plant material.

For the evaluation of the chromatographic profile of selected medicinal plants extracts, the polyphenolic and volatile oil constituents considered the most widespread secondary metabolites in the medicinal plants were separated using optimized HPTLC conditions. To enhance the separation process, the plate was developed in two steps using different mobile phases. According to the selectivity of the mobile phase used in the first step (ethyl acetate–formic acid–water mixture) highly and medium polar phenolics were accurately separated as individual bands with well-defined shapes. The mobile phase used for the second development step (toluene–ethyl acetate) was selected to separate only the lipophilic compounds, positioned on the front line of the first development. As a result of the second development there would be complementary separation of the lipophilic compounds from the hydrophilic ones. Some advantages can also be obtained by using the double development approaches and the same separation condition for all extracts. The first refers to double development with different mobile phases which provide a greater separation capacity of the compounds and allow a better highlight of the similarities and differences between the chromatographic fingerprints. The second advantage would be related to the creation of a reference fingerprint database for species identification that requires the same chromatographic conditions for all the analyzed samples. The visual examination of the HPTLC chromatograms obtained under multiwavelength detection using 254 nm and 365 nm, respectively, revealed a different pattern in chemical composition of the analyzed extracts (Figure S1, supplementary material). Detection under 365 nm revealed that these extracts are rich in phenolic compounds with a pattern dominated by blue, red and yellow-orange color bands. 

By applying the second development step, the separation of new compounds was revealed. This separation brings additional information to the chromatographic fingerprints of the analyzed extracts. Thus, complementary information can be obtained from the chromatographic profile after single and double development of the plate. Moreover, multiwavelength detection applied after the second development using the fluorescence quenching at 254 and fluorescence at 365 nm (before and after selective spraying of the plate by specific derivatization reagents) (Figure S1) also reveals complementary information on the fingerprint profiles.

### 3.2. Evaluation of Color Scales HPTLC Fingerprints

**Figure 1:**
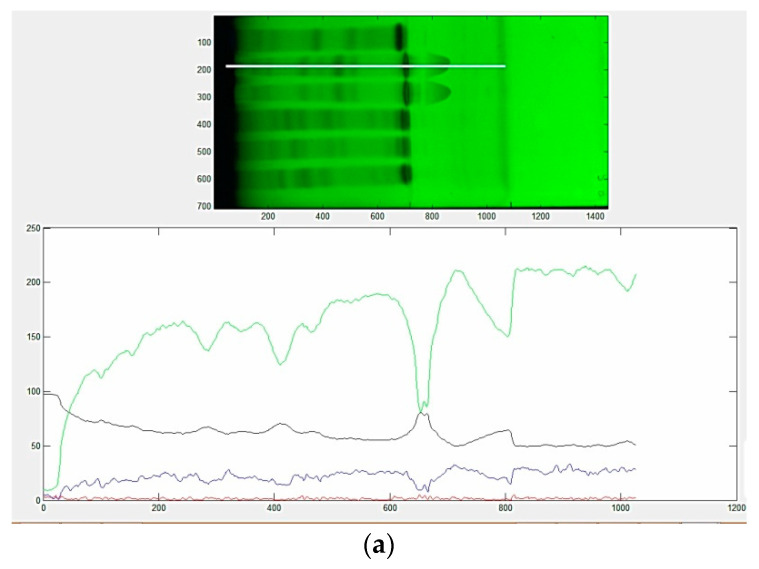

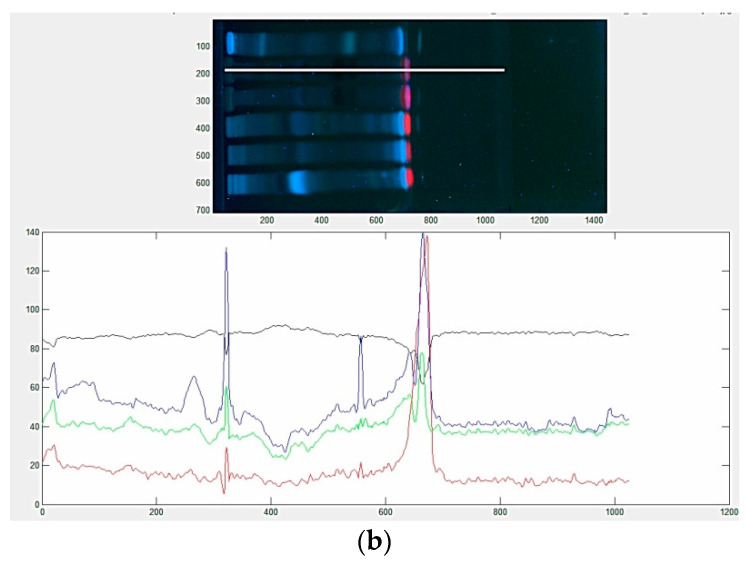
Image of chromatographic plate after the second development and corresponding fingerprints for *Juniperus communis* L. (JuM) using TLC Analyzer software and different color scale selection in: (**a**) fluorescence quenching mode under 254 nm; (**b**) fluorescence mode under 365 nm excitation wavelength. Red line—red scale fingerprint (R); green line—green scale fingerprint (G); blue line—blue scale fingerprint (B); gray line—gray scale fingerprint (K).

To differentiate and/or confirm the identity of a sample, a comparative evaluation of the chromatographic fingerprint can be made using a reference material. The main problem is the natural variance of plants. From this point of view, a fingerprint should enable accurate identification of the plant material even if the concentrations of the marker compounds are slightly different from reference plant material and should also be able to demonstrate the uniformity and the differences between several samples. Colorful HPTLC chromatograms from multiwavelength imaging of the HPTLC plates combined with the splitting of images through gray (K), red (R), green (G) and blue (B) channels increase selectivity and offer a huge amount of additional information differentiating compounds according to their fluorescent colors. For complex mixtures with differently absorbing compounds, as was revealed for the analyzed extracts, multiwavelength imaging combined with different color scale approaches is beneficial because they can reveal the presence of various classes of compounds. In order to extract the maximum useful information from the chromatographic plate, the images of HPTLC plates acquired under UV 254 nm and 365 nm, respectively, were processed via TLC Analyzer software. Color scale fingerprints (Figure 1), describing changes in the optical density and intensity of the signal proportional to the concentration of mixture components for images acquired under UV 254 nm and 365 nm, respectively, were used in this study as variables for the chemical profile analysis. Under 254 nm detection, the green channel revealed a higher sensitivity while more or less similar profiles were observed for detection under 365 nm.

The color intensity values from start to front distance for each of the analyzed samples from every of the digitized R, G, B and K, color scale fingerprint (digitized chromatogram, matrix of 39 samples × 681 variables after one development step (D1) and 39 samples × 1041 variables after two development steps) were further used in PCA and CA analysis of the samples.

### 3.3. Chemometric Analysis of the Color Scale Fingerprints

The evaluation of the samples profiles in a single run requires the application of chemometric methods in order to extract the maximum useful information. For this, multivariate analysis methods were applied on data obtained by digitization of red, green, blue and gray color scale (R, G, B and K) fingerprints acquired with TLC Analyzer software.

**Figure 2:**
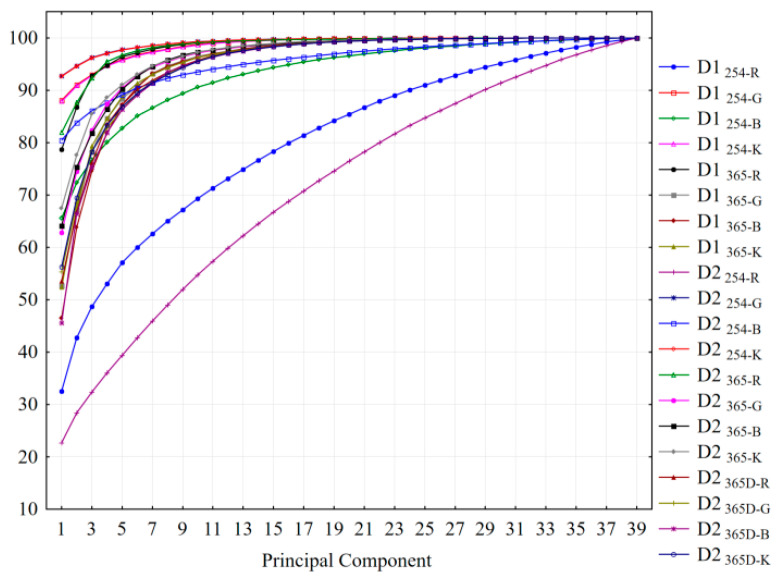
The profiles of cumulative proportion corresponding to the first PCs accounting 100% of the total variance: D1—one development of the plate; D2—double development of the plate; 254—detection under 254 nm; 365—detection under 365 nm; 365D—detection under 365 nm after selective spraying of the plate by specific derivatization reagents; R—red scale selection; G—green scale selection; B—blue scale selection; K—gray scale selection.

The PCA profiles based on data from digitized color scale fingerprints (Figure 2) revealed that in almost all of the cases, the first three PCs account for more than 75% of data variability. The exception was observed for red scale fingerprints (R) obtained under 254 nm documentation where the first component (PC1) accounts for the smallest proportion in both cases using one and double development of the chromatographic plate (22.48% for one development—D1 and 22.70% in case of double development—D2).

**Figure 3:**
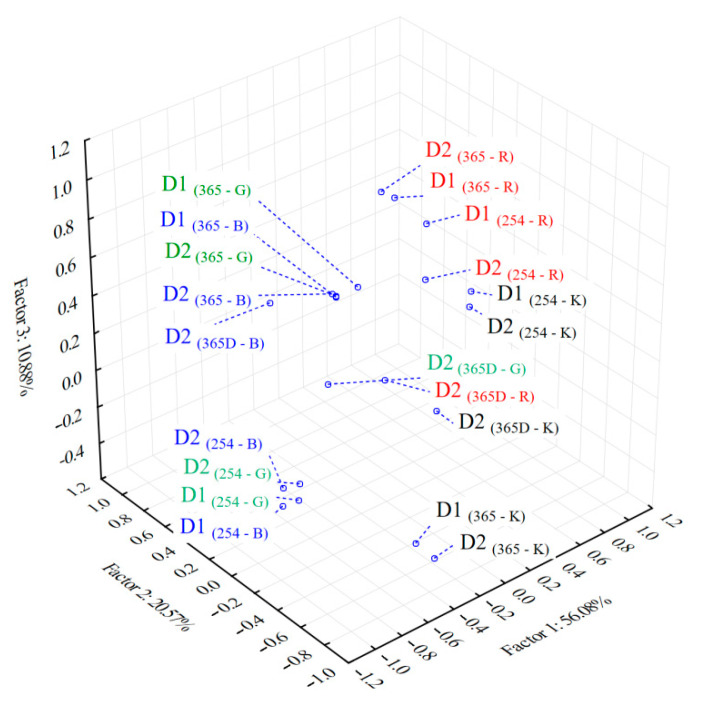
3D PCA classification of the color scales fingerprints (R—red, G—green; B—blue; K—gray) based on specific chemical profiles of the analyzed extracts: D1—one development of the plate; D2—double development of the plate; 254—detection under 254 nm; 365—detection under 365 nm; 365D—detection under 365 nm after selective spraying of the plate by specific derivatization reagents of chromatographic plate after the second development and digitised chromatograms for *Juniperus communis* L. (JuM) corresponding to different color scale selection obtained by processing the UV fingerprints using TLC Analyzer software.

The factor loadings after Varimax rotation of the PCs obtained in PCA analysis of data from both developments and all the color scale fingerprints (39 samples × 780 variables (PCs) including all the four channels, both development steps and three modes of plate documentation) showed that the first three factors describe over 87% of total variance of the initial data (Table 2). The patterns obtained by 3D projection of the scores of the first factors show the grouping of the color scales according to their similar contribution to the specific chemical profiles in the analyzed samples (Figure 3). As a general observation the green (G) and blue (B) scale fingerprints reveal similar chemical profiles in both wavelength imaging detection modes, under 254 nm and 365 nm, respectively. A similar chemical profile was revealed also in case of red (R) and gray (G) color scale fingerprints for imaging at 254 nm. The use of imaging at 365 nm wavelength after plate derivatization with specific reagents (D2-365D) revealed a different chemical profile from blue scale (B) compared to red (R), green and gray (K) scales.

According to the factor loadings values (Table 2) the first factor (Factor 1, 56% contribution) is associated with the information provided by the first and second development of the plate and visualization under 254 nm. The visualization at 365 nm before and after derivatization also brings important information that is revealed by the high contribution of the second factor (Factor 2, 20%). A strong contribution (loadings > 0.93) of the green, blue and gray (G, B, K) color scale fingerprints was revealed for both wavelength imaging (under 254 nm and 365 nm, respectively) and also for single and double development of the chromatographic plate. An improved contribution was generally observed in all cases after the second development from color scale fingerprints obtained from images under 365 nm.

Blue scale (B) fingerprint was revealed with a strong contribution (loading value of 0.94) to the chemical profiling of the samples after the second development and selective spraying of the plate with specific derivatization reagents (D2 _(365D-B)_). Red scale (R) fingerprints from images of double and respectively one development of the plate revealed a strong contribution (loading values of 0.98 and 0.94) in case of plate documentation under 365 nm.

The use of the color scale fingerprints information for a complete evaluation of the chromatographic profile of complex samples was recently proposed in literature [38] where good results were obtained for classification of a set of medicinal plants (belonging to a single region) according to their phylum. Based on the factor analysis results, it can be concluded that the use of chemical profiles after one and double development respectively combined with multiwavelength imaging and color scale fingerprinting approach allow the extraction of complementary or useful information from the chromatographic plate and can improve the classification processes of complex samples as medicinal plant extracts. 

### 3.4. Hierarchical Clustering Analysis of Medicinal Plants Extracts

The hierarchical cluster analysis (HCA) with joining tree clustering algorithm was used for identification of characteristic clusters based on the similarities in the chemical profile of samples revealed by multiwavelength imaging and color scales fingerprinting analysis. The main advantage of HCA is the flexibility to alter the similarity measurement criterion and the applied linkage method to suit different applications.

**Figure 4:**
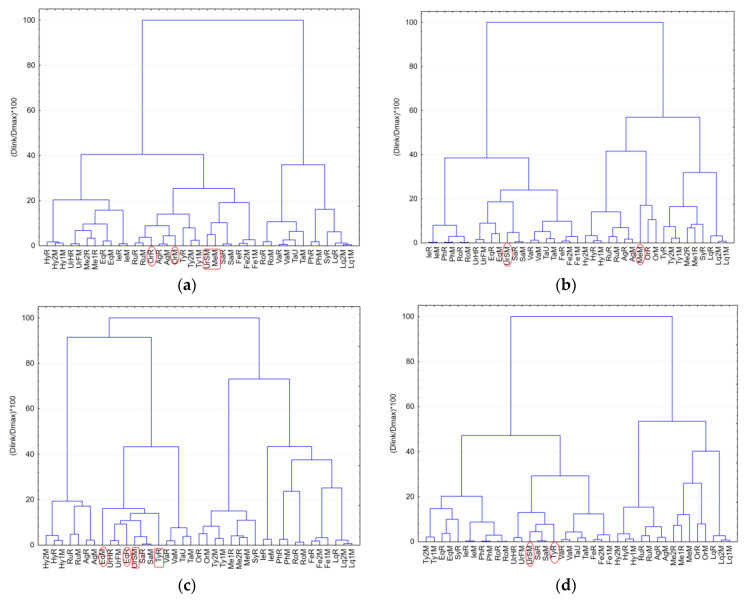
Classification of the medicinal plant extracts based on color scale fingerprints data from multiwavelength imaging (under 254 nm and 365 nm detection) using the cluster analysis (CA) technique based on 1-Pearson distance measurement with Ward’s amalgamation method: (**a**) using data from red scale (R) fingerprints; (**b**) using data from green scale (G) fingerprints; (**c**) using data from blue scale (B) fingerprints; (**d**) using data from gray scale (K) fingerprints.

By a visual inspection of the clusters obtained for individual color scale fingerprints data (R, G, B and K, respectively), in all cases the best classification of the samples with a meaningful interpretation of clustering results was obtained using 1-Pearson distance measurement with Ward’s amalgamation (linkage) method. Using data from both single and double development of the plate (D1 and D2) and from multiwavelength imaging analysis (254 nm, 365 nm and 365 nm after specific derivatization) different chemical patterns were revealed for some of the analyzed samples depending on the used color scale fingerprint (Figure 4).

The red scale (R) fingerprints revealed differences in the chemical composition (samples not closely clustered) for *Origanum vulgare* L. (Or) samples of different provenience while blue scale (B) fingerprint revealed a different chemical profile for *Equisetum arvense* L. (Eq) from Romania and Macedonia. *Urtica dioica* L. seeds sample (UrSM) was revealed with a different chemical composition profile by all color scale fingerprint. Blue (B) and gray (K) scale fingerprints differentiate the *Thymus serpyllum* L. samples from Romania while green (G) and red (R) scales fingerprints highlighted a different chemical profile for *Mentha piperita* L. from Macedonia (MeM). 

**Figure 5:**
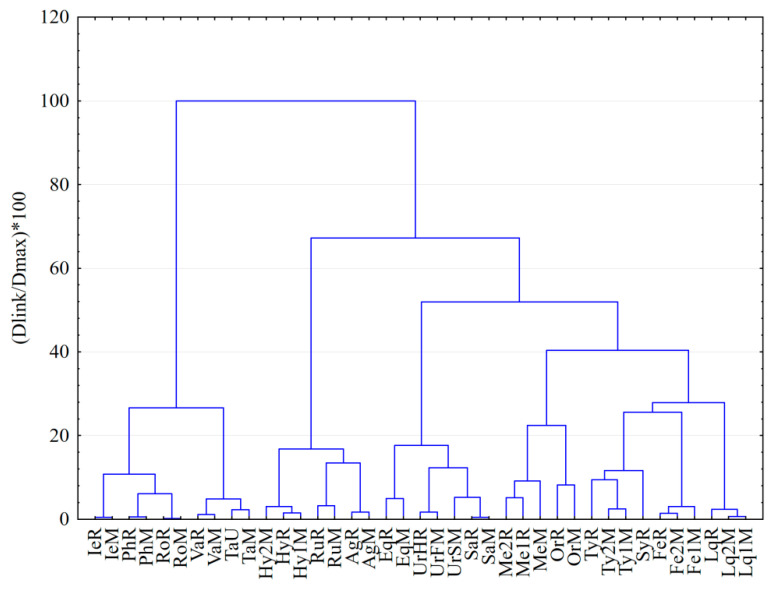
Dendrogram of the medicinal plant extracts grouped by hierarchical clustering method (HCA) using data from red (R), blue (B), green (G) and gray (K) color scale fingerprints, multiwavelength imaging (under 254 nm and 365 nm detection before and after derivatization) and both development steps (D1, D2) applying 1-Pearson distance measurement with Ward’s amalgamation method.

Result applying HCA on data from all the color scales fingerprints (R, G, B and K) reveal the samples to split in clusters with considerable level of similarity (direct linkage at low level) for the same medicinal plant species regardless the region of provenience and storage conditions (Figure 5). Similar HCA dendrograms were obtained applying the same chromatographic conditions and image analysis protocol for duplicate chromatographic experiments. In all cases the clusters grouping samples from the same species are linked based on minimum 88% similarity in their chromatographic fingerprint. Stinging nettle samples are grouped together, but in two different subclusters revealing a different composition of the seeds (UrSM) comparative with the folium (UrFM), respectively, the herba aerial part (UrHR) of the plant.

Based on the above results, the use of data from color scale fingerprints from double development procedure and multiple visualization modes combined with HCA analysis using 1-Pearson distance measurement with Ward’s amalgamation (linkage) method proved to detect the similar medicinal plant extracts even though they are from different geographical regions, have different storage conditions and no specific markers are individually extracted. The proposed approach could be a promising tool for authentication and identification studies of plant materials based on HPTLC fingerprinting analysis. 

## 4. Conclusions

The advantages of the use of multiwavelength imaging and color scale HPTLC fingerprints for clustering of medicinal plants extracts were evaluated by chemometric approaches. The complementary information from double development of the plate combined with the UV 365 nm detection was revealed to better characterize the chemical profile of samples. The advantages of double development of the plate and significant contribution of each of the color scale (red, green and blue and respectively grey) fingerprints on the characterization of the chemical profile of complex samples was revealed by use of principal component analysis with factor analysis methods (PCA-FA). Under 254 nm and respectively 365 nm wavelength imaging, green (G) and blue (B) scale fingerprints have been identified to bring similar information related to the chemical profiles of samples. Red (R) and gray (G) color scale fingerprints provide similar information for imaging at 254 nm. Moreover, blue scale (B) fingerprint was revealed to be more informative for characterization of the chemical profile after derivatization of the separated compounds with specific reagents and 365 nm wavelength imaging. The use 1-Pearson distance measurement with Ward’s amalgamation in the HCA analysis of data from the multiwavelength imaging and all the color scale fingerprints proved to allow a better evaluation of the similarities/differences in chemical pattern of complex samples and easy visualization of their clustering. Taking into account all the findings, it was possible to classify and identify the medicinal plants according to their species regardless of the geographical regions of provenance. Using this approach, a new protocol can be proposed for further investigation in HPLTC fingerprinting and direct identification and authentication of medicinal plant extracts. 

## Figures and Tables

**Table 1:** Common/scientific name for the investigated medicinal plants.

No.	Common Name	Genus (Taxonomic Rank)	Familly	Part/Plant	Code Label ^1^	Provenience	Herbarium No.
1	Liquorice	*Glycyrrhiza glabra* L.	Fabaceae	Radix	Lq1M	North Macedonia	1/2018
					Lq2M	North Macedonia	2/2018
					LqR	Romania/Fares	22/2018
2	Sweet fennel	*Foeniculum vulgare var.dulcis* Mill.	Apiaceae	Fructus	Fe1M	North Macedonia	3/2018
					Fe2M	North Macedonia	4/2018
					FeR	Romania/Dacia Plant	23/2018
3	Dandelion	*Taraxacum officinale* F.H.Wigg.	Asteraceae	Radix	TaM	North Macedonia	5/2018
					TaU	Hungary/ Gyogyfu KFT	39/2018
4	Valerian	*Valeriana officinalis* L.	Caprifoliaceae	Radix	ValM	North Macedonia	6/2018
					ValR	Romania/	24/2018
5	Dog rose	*Rosa canina* L.	Rosaceae	Fructus	RoM	North Macedonia	7/2018
					RoR	Romania/Fares	25/2018
6	Comfrey	*Symphytum officinale* L.	Boraginaceae	Radix	SyR	Romania/Fares	26/2018
7	Juniper	*Juniperus communis* L.	Cupresaceae	Fructus	JuM	North Macedonia	8/2018
					JuR	Romania/Plafar	27/2018
8	Wild thyme	*Thymus serpyllum* L.	Lamiaceae	Herba	Ty1M	North Macedonia	9/2018
					Ty2M	North Macedonia	10/2018
					TyR	Romania/Plafar	28/2018
9	Bean	*Phaseolus vulgaris* L.	Fabaceae	Pericarpum	PhM	North Macedonia	11/2018
					PhR	Romania/Fares	29/2018
10	Elderberry	*Sambucus nigra L.*	Adoxaceae	Flowers	SaM	North Macedonia	12/2018
					SaR	Romania/Fares	30/2018
11	Horsetail	*Equisetum arvense* L.	Equisetaceae	Herba	EqM	North Macedonia	13/2018
					EqR	Romania/Fares	31/2018
12	Oregano	*Origanum vulgare* L.	Lamiaceae	Herba	OrM	North Macedonia	14/2018
					OrR	Romania/Fares	32/2018
13	Agrimony	*Agrimonia eupatoria* L.	Rosaceae	Herba	AgM	North Macedonia	15/2018
					AgR	Romania/Fares	33/2018
14	Blackberry	*Rubus fruticosus* L.	Rosaceae	Folium	RuM	North Macedonia	16/2018
					RuR	Romania/Plafar	34/2018
15	Peppermint	*Mentha x piperita* L.	Lamiaceae	Folium	MeM	North Macedonia	17/2018
				Herba	Me1R	Romania/Fares	35/2018
				Folium	Me2R	Romania/Plafar	36/2018
16	St.John’s wort	*Hypericum perforatum* L.	Hypericaceae	Herba	Hy1M	North Macedonia	18/2018
					Hy2M	North Macedonia	19/2018
					HyR	Romania/Fares	37/2018
17	Stinging nettle	*Urtica dioica* L.	Urticaceae	Seeds	UrSM	North Macedonia	20/2018
				Folium	UrFM	North Macedonia	21/2018
				Herba	UrHR	Romania/Fares	38/2018

^1^ M—Medicinal plants from North Macedonia; R—Medicinal plants from Romania; U—Medicinal plants from Hungary.

**Table 2:** Factor loadings after Varimax rotation of the first three PCs with eigenvalue > 1.0 reflecting the contribution of each color scale in grouping the samples according to the information provided by UV (254 nm and 365 nm) fingerprints (marked loadings are >0.700)/scientific name for the investigated medicinal plants.

Variables *	Factor 1	Factor 2	Factor 3
D1 _(254-R)_	0.46	0.17	0.68
D1 _(254-G)_	**−0.94**	−0.30	−0.14
D1 _(254-B)_	**−0.93**	−0.15	−0.24
D1 _(254-K)_	**0.94**	0.29	0.14
D1 _(365-R)_	0.14	0.09	**0.94**
D1 _(365-G)_	0.52	**0.83**	0.07
D1 _(365-B)_	0.4	**0.89**	0.04
D1 _(365-K)_	−0.51	**−0.80**	−0.30
D2 _(254-R)_	0.64	0.37	0.26
D2 _(254-G)_	**−0.95**	−0.31	−0.05
D2 _(254-B)_	**−0.96**	−0.19	−0.12
D2 _(254-K)_	**0.95**	0.31	0.04
D2 _(365-R)_	0.07	0.13	**0.98**
D2 _(365-G)_	0.41	**0.9**	0.03
D2 _(365-B)_	0.38	**0.9**	0.05
D2 _(365-K)_	−0.38	**−0.81**	−0.42
D2 _(365D-R)_	−0.04	−0.02	0.09
D2 _(365D-G)_	−0.11	0.39	−0.08
D2 _(365D-B)_	−0.08	**0.94**	0.13
D2 _(365D-K)_	0.09	−0.32	0
**Total variance (%)**	**56.08**	**20.57**	**10.88**
**Cumulative variance (%)**	**56.08**	**76.66**	**87.53**

* D1—one development of the plate; D2—double development of the plate; 254-R, G, B and K—red, green, blue and gray scale for fingerprint digitization of images of the HPTLC plates obtained under UV documentation at 254 nm; 365-R, G, B and K—red, green, blue and gray scale for fingerprint digitization of images of HPTLC plates obtained under UV documentation at 365 nm. 365D-R, G, B and K—red, green, blue and gray scale for fingerprint digitization of images of HPTLC plates obtained under UV documentation at 365 nm after derivatization procedure.

## Data Availability

The data presented in this study is available upon request from the corresponding author.

## Supplementary Materials

The following are available online. Figure S1: The images of the chromatographic plates (HPTLC Silica gel 60 F_254_ plates) including all the analysed medicinal plants extracts obtained after first development (D1) using ethyl acetate–formic acid–water (80:10:10 *v/v/v*) as mobile phase and second development (D2) using toluene–ethyl acetate (95:5, *v/v*) as mobile phase; visualization by fluorescence quenching under 254 nm (a), florescence at 365 nm (b), and at 365 nm after derivatization (c).

## Sample Availability

Not available.
