# Supplementary material for: Application of HPTLC Multiwavelength Imaging and Color Scale Fingerprinting Approach Combined with Multivariate Chemometric Methods for Medicinal Plant Clustering According to Their Species
Source: Molecules. 2021 Nov 29;26(23):7225. doi: 10.3390/molecules26237225 (PMC8659119; doi:10.3390/molecules26237225)
Supplement: Supplementary file 1 [file molecules-26-07225-s001.zip › molecules-1434483-supplementary.pdf]

Application of HPTLC multi-wavelength imaging and color scale fingerprinting approach combined with multivariate chemometric methods for medicinal plant clustering according to their species

Simona Codruța Aurora Cobzac [1,2] , Neli Kinga Olah [3,4], Dorina Casoni [1,2,*]

[1] Babeş-Bolyai University, Faculty of Chemistry and Chemical Engineering, Department of Chemistry, Arany János, No. 11, Cluj-Napoca, România

[2] Research Center for Advanced Chemical Analysis, Instrumentation and Chemometrics – ANALYTICA, Babeş-Bolyai University 11 Arany Janos str., RO-400028, Cluj-Napoca, Romania

[3] "Vasile Goldis" Western University of Arad, Faculty of Pharmacy, 86 L. Rebreanu str., Arad, Romania

[4] SC PlantExtrakt SRL, 407059 Rădaia, Cluj, Romania

* Correspondence: dorina.casoni@ubbcluj.ro; Tel.: +40-264-590818

Plate number	
1	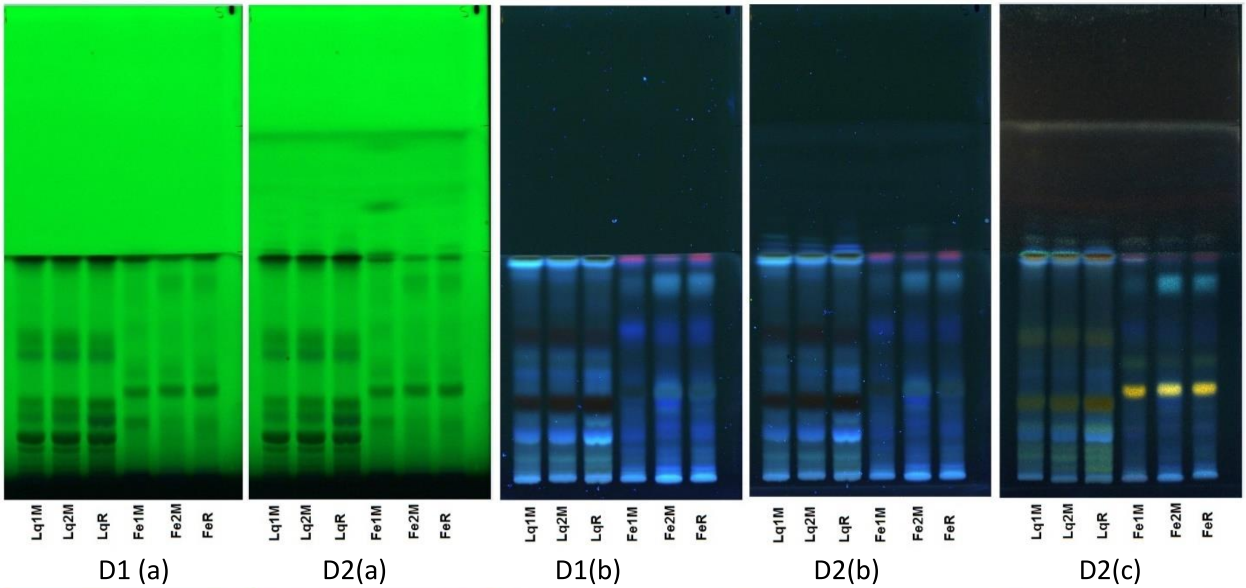
2	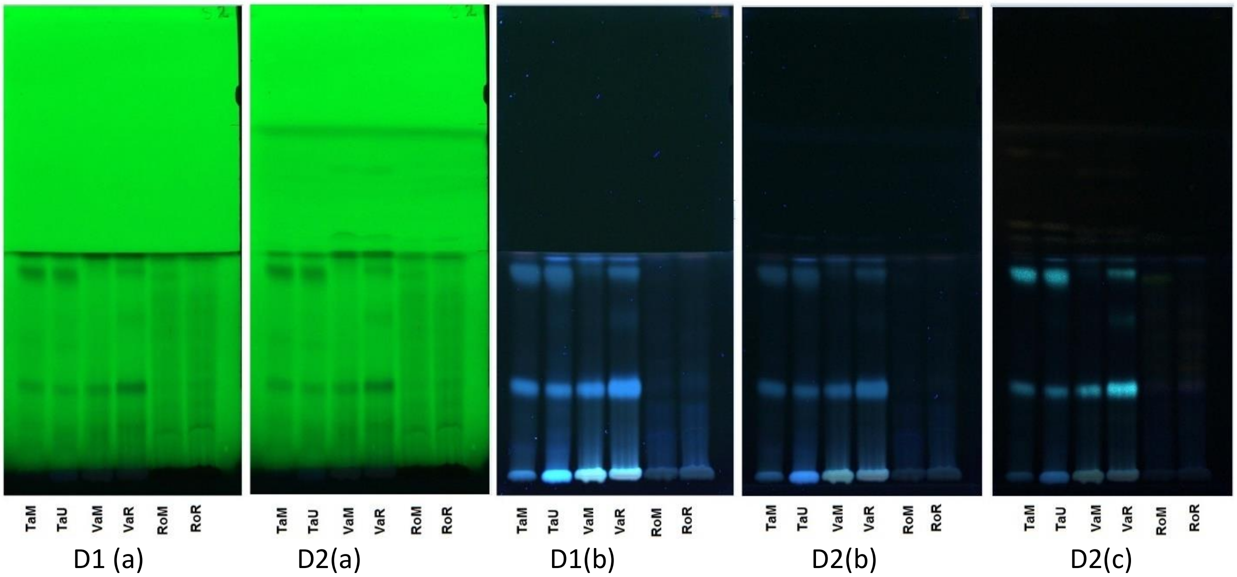

3

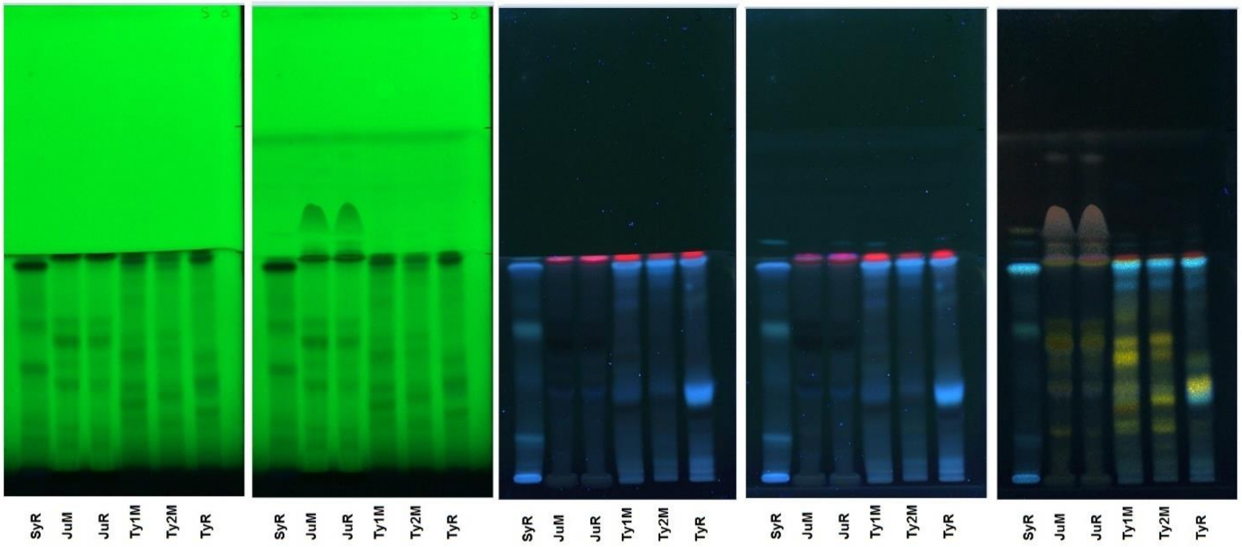

D1 (a) D2(a) D1(b) D2(b) D2(c)

4

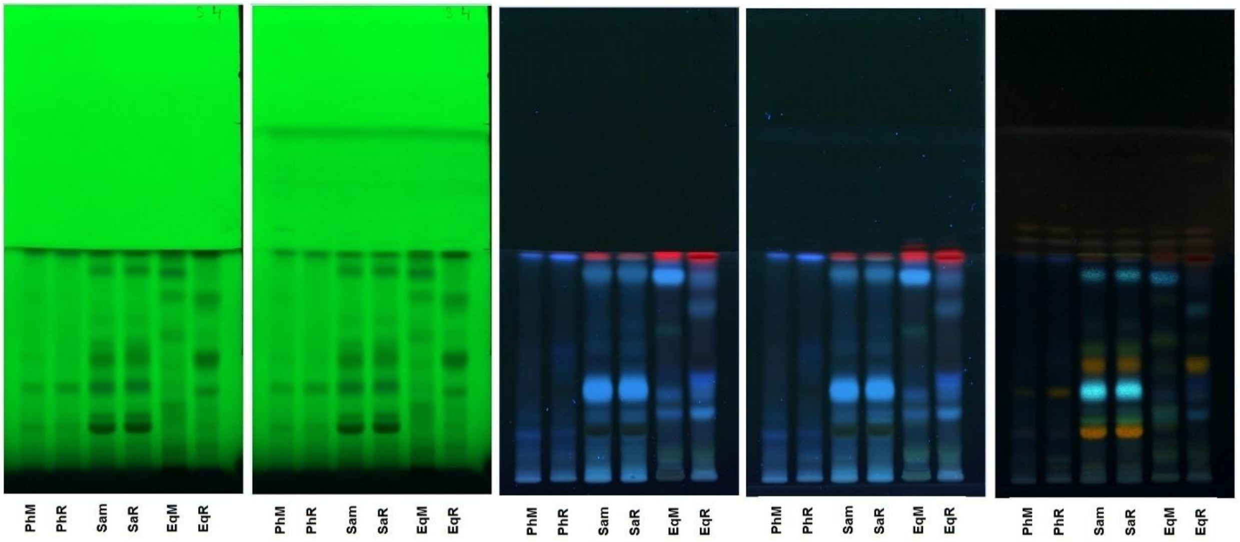

D1 (a) D2(a) D1(b) D2(b) D2(c)

5

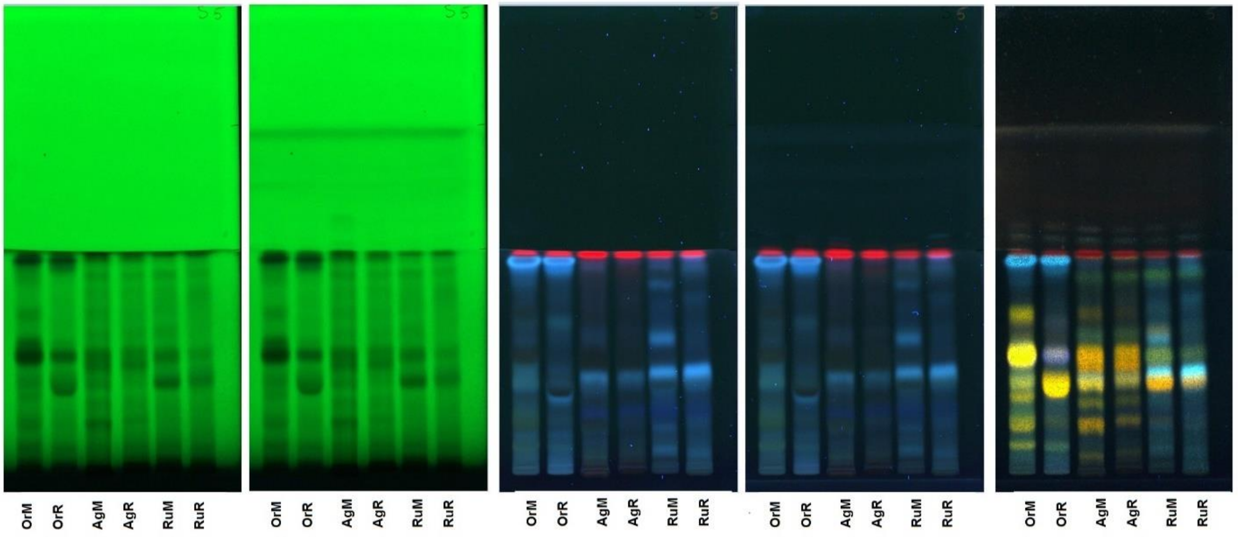

D1 (a) D2(a) D1(b) D2(b) D2(c)

6

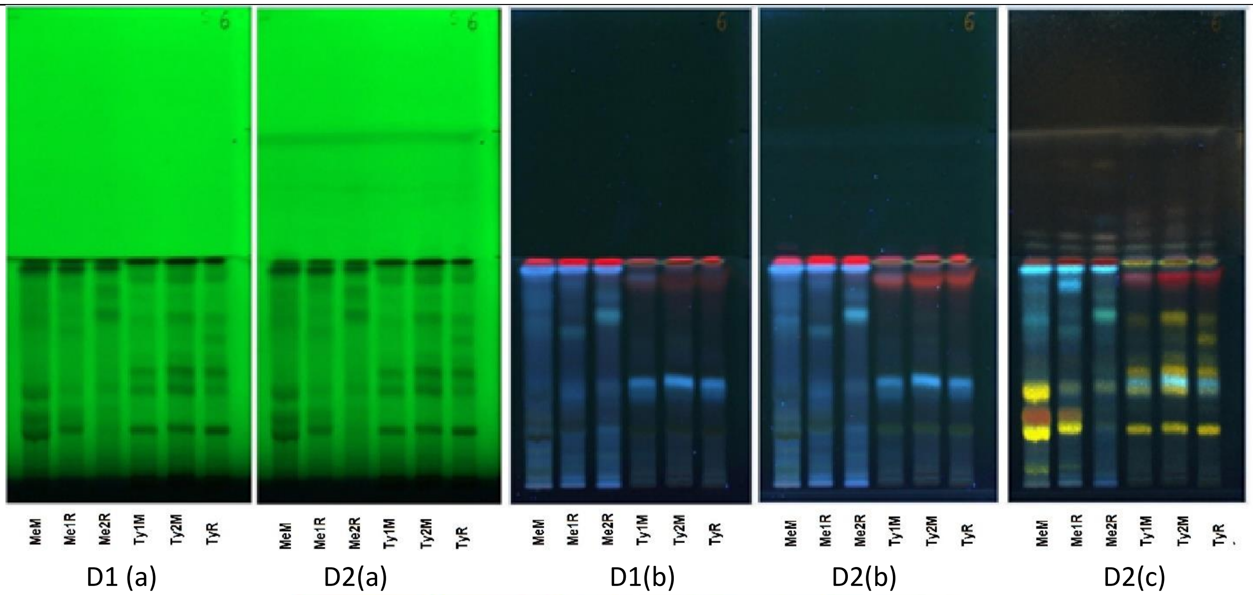

7

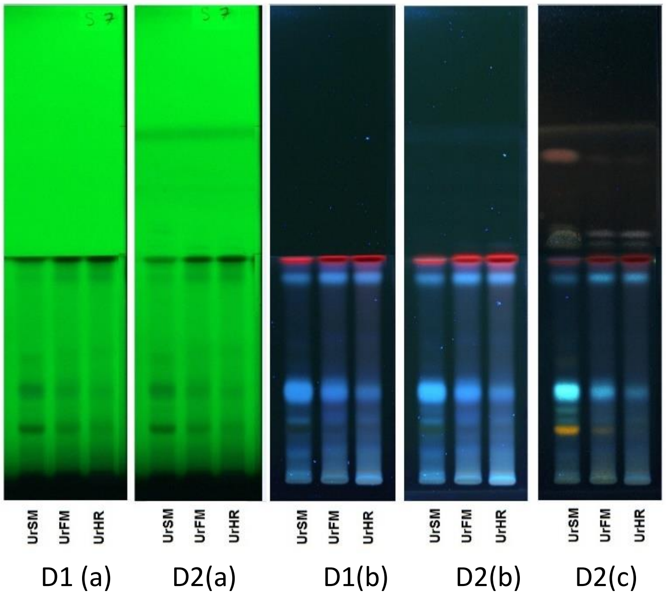

Figure S1. The images of the chromatographic plates (HPTLC Silica gel 60 F_{254} plates numbered from 1 to 7) including all the analyzed medicinal plants extracts (corresponding name of the plants - in Table 1) obtained after first development (D1) using ethyl acetate - formic acid - water (80:10:10 v/v/v) as mobile phase and second development (D2) using toluene - ethyl acetate (95: 5, v/v) as mobile phase respectively; visualization (from left to right side) by fluorescence quenching under 254 nm (a), florescence at 365 nm (b)and at 365 nm after derivatization (c).
